# A rare association of arteriovenous malformation of the omentum and pseudo-Meigs’ syndrome: case report and scoping review of literature

**DOI:** 10.1093/jscr/rjad080

**Published:** 2023-03-15

**Authors:** Mazin S Baazeem, Modhi M AlJumah, Norah F AlSalim, Salman AlMalki

**Affiliations:** Department of Obstetrics and Gynecology, Women Specialized Hospital, King Fahad Medical City, Riyadh, Saudi Arabia; King Khalid University Hospital, King Saud University Medical City, Riyadh, Saudi Arabia; King Saud University, Riyadh, Saudi Arabia; Department of Pathology and Clinical Laboratory Medicine, King Fahad Medical City, Riyadh, Saudi Arabia

**Keywords:** Pseudo-Meigs’ Syndrome, Leiomyoma, Arteriovenous malformation

## Abstract

Meigs’ syndrome is defined as a secondary triad of ascites, pleural effusion and benign ovarian tumor, usually fibroma. While pseudo-Meigs’ syndrome is a rare condition that is associated with benign ovarian tumor—other than fibroma—or even malignant. The case presented is a 40-year-old Saudi, nulliparous woman who was referred for precise diagnostic work-up as a case of huge pelvic-abdominal mass, tense ascites and pleural effusion. After further investigations cancer antigen-125 was found to be elevated. An abdominal CT scan revealed significant interval increase in the size of ascites, which cause huge abdominal distention, as well as a significant pleural effusion. Pathology of surgical specimens revealed a giant uterine leiomyoma, whereas the omentum excision surprisingly confirmed multiple disorganized arteries and veins, which resulted in omental arteriovenous malformation. To the best of our knowledge, this is the first reported case in the worldwide literature of two different rare conditions.

## INTRODUCTION

Meigs’ syndrome is a rare medical condition that is characterized by the presence of a benign ovarian tumor usually a fibroma, in combination with accumulation of fluid in the abdomen called ascites and the buildup of fluid in the pleural cavity called pleural effusion [1]. Pseudo-Meigs’ syndrome is an unusual condition that resembles Meigs’ syndrome, but it differs in that it is not caused by a fibroma. Instead, it is caused by other benign ovarian tumors, malignant ovarian tumors either primitive tumor or ovarian metastasis from another primitive tumor or non-ovarian tumor, i.e. fallopian tubes or uterus [2, 3]. Symptoms of pseudo-Meigs’ syndrome are similar to those of Meigs’ syndrome, including abdominal swelling, abdominal discomfort, shortness of breath and weight gain. Symptoms will be resolved immediately by the resection of the tumor in both of them [4]. Although serum cancer antigen-125 (CA-125) is not usually elevated, it raises the suspicion of malignant ovarian tumors particularly in postmenopausal women with a solid adnexal mass. Despite the high likelihood of malignancy, a benign disease like Meigs’ syndrome cannot be excluded because of similarity in their presentation [5]. Apart from this, the omentum is a layer of fatty tissue that surrounds the organs in the abdomen and the arteriovenous malformation (AVM) of the omentum is a very rare condition in which there are abnormal connections or passageways between the arteries and veins in the omentum. The clinical consequences always result from rupture and hemorrhage [6]. Typically, AVMs are present at birth but may not be clinically evident. During childhood, they particularly become evident and frequently exacerbated during puberty or pregnancy [7].

## CASE PRESENTATION

**Figure 1:**
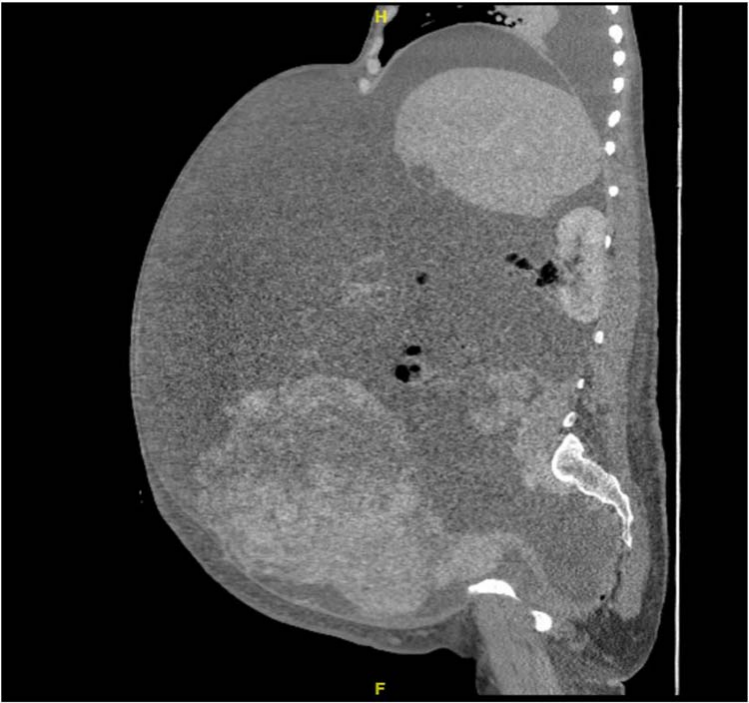
Abdominal CT scan shows huge ascites.

**Figure 2:**
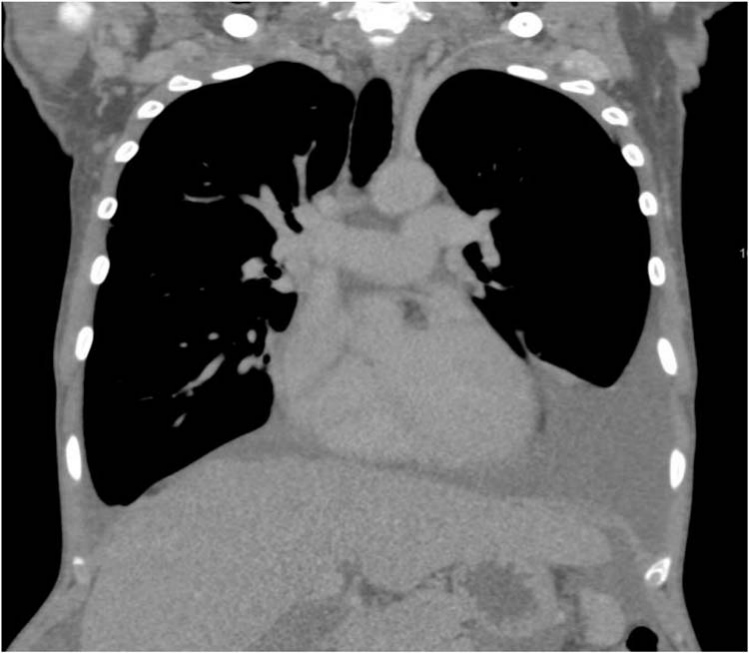
CT scan of the chest shows pleural effusion.

**Figure 3:**
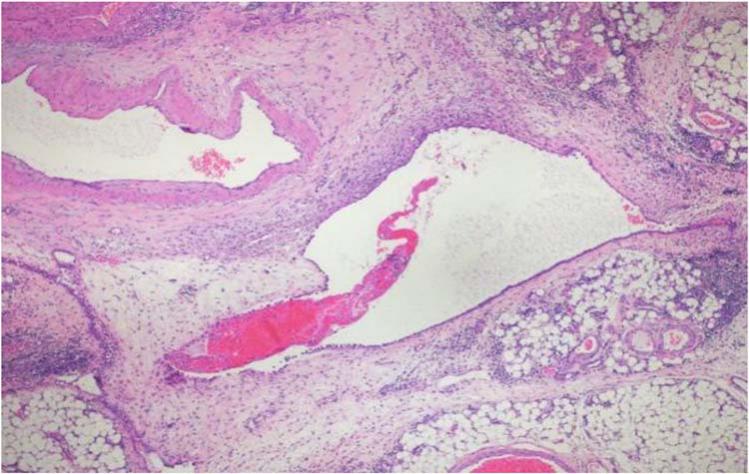
H&E stain of the omentum excision shows multiple, large and irregular disorganized veins and arteries.

**Figure 4:**
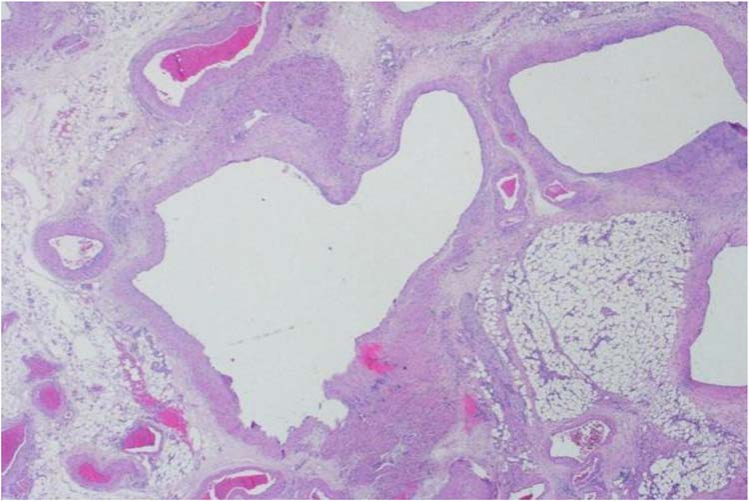
H&E stain of the omentum excision shows multiple, large and irregular disorganized veins and arteries.

A 40-year-old Saudi, nulliparous woman, medically free with a history of leiomyoma (uterine fibroid) underwent myomectomy. She was referred to our center as a case of large abdominopelvic mass, tense ascites and pleural effusion. The patient complained of the shortness of breath, palpitation, marked abdominal discomfort and bloating as well as back pain. She reported that she first noticed the bloating 3 months prior to the visit. However, she was unable to come because of COVID-19 pandemic situation. She is a housewife; never smoked tobacco or drank alcohol, and had no history of recent travel to endemic or pandemic areas. On further investigations, a serum CA-125 was found to be elevated 492.6 U/mL (normal < 35 U/mL), whereas the concentration of other tumor markers LDH, CEA, CA19-9 and ALP was within normal ranges. Abdominal CT scan revealed a significant interval increase in the size of ascites, which caused a huge abdominal distention along with severe mass effects on the abdominopelvic organs, which have been displaced posteriorly as shown in Fig. 1. In addition, it revealed a large exophytic uterine fibroid measuring 15 × 20 × 17.5 cm. A therapeutic paracentesis was done with the removal of 50.750 ml of ascitic fluid. Figure 2 shows how pleural effusion is markedly significant. A total abdominal hysterectomy, with bilateral salpingo-oophorectomy and omentectomy, was performed. Intraoperative finding revealed a very large mass, which originated from uterine fundus. The pleural effusion and ascites disappeared entirely after the excision of abdominopelvic mass. She recovered completely and went through an uneventful postoperative period. On pathology of surgical specimens, a giant uterine leiomyoma with degenerative changes, pleural and ascitic fluids was yielded negative for malignancy and diagnosed as a benign mesothelial proliferation, whereas H&E stain of the omentum excision surprisingly confirmed multiple, large and irregular disorganized veins (thin-walled blood vessels) and arteries (thick-walled blood vessels), some with organized thrombi consistent with omental AVM as shown in Figs 3 and 4. Provisional diagnosis of pseudo-Meigs’ syndrome with omental AVM was considered.

## DISCUSSION

Omental lesions have a difficult preoperative diagnosis and the diagnosis is often made postoperatively. Accurate diagnosis is made through the histopathological examination of the lesion [8]. In our case, she has AVM with omental involvement. AVM of the omentum is extremely rare, only one case of AVM of the omentum in a 5-year-old girl was reported [9]. The etiology is not yet clear. No study reported in the literature up to date. Pseudo-Meigs’ syndrome is a rare condition that is associated with secondary accumulation of ascites and pleural effusion with a pelvic mass other than fibroma [10]. In our case, the patient has pseudo-Meigs’ syndrome that is caused by uterine leiomyoma. A pseudo-Meigs’ syndrome originating from uterine leiomyoma is extremely rare, only few cases have been reported on patients with uterine leiomyoma presenting as a case pseudo-Meigs’ syndrome [11]. Although the epidemiology of pseudo-Meigs’ syndrome is unknown. Uterine fibroids are very common, with about 70% of women developing them at some point in their lives [8]. Because of the similarity in symptoms between ovarian cancer, Meigs’ syndrome and pseudo-Meigs’ syndrome, they are often confused together at first sight [5]. Only about 1% of all ovarian tumors present as Meigs’ syndrome [12]. They are rarely seen as well as their pathophysiology remain not very well understood [13]. Furthermore, Pseudo-Meigs’ syndrome typically occurs in postmenopausal women with an average age of 50-year-olds [12]. Elevated serum CA-125 in postmenopausal women usually increases the suspicion of malignant conditions. However, it is not a specific indicator and can be elevated in many benign conditions, for instance: pelvic inflammatory disease, pregnancy, endometriosis, cirrhosis, benign ovarian tumors and uterine leiomyomas [14]. In the literature review of 23 studies, we found that the CA-125 increases with pseudo-Meigs’ syndrome as well. In our case, the CA-125 was significantly elevated and she had a radiological appearance that mimicked a malignant tumor. To the best of our knowledge, this is the first reported case of two different rare conditions that combined omental AVM and gynecologic oncological issue. Finally, there is general lack in literature regarding AVM in older age groups especially women, omental vascular malformation, its association with gynecological tumors and parity status as well.

## CONCLUSION

Omental AVM is an extremely rare lesion that could have significant presentation. A high index of suspicion for pseudo-Meigs’ syndrome should be entertained in women presenting with abdominopelvic mass, ascites and pleural effusion with benign clinical features especially for those with previous history of uterine fibroids. This case report raises questions for further discourse and research on the omental AVM and its association with pseudo-Meigs’ syndrome.

## Data Availability

The authors confirm that the data supporting the findings of this study are available within the article. Raw data that support the findings are available from the corresponding author upon reasonable request.
